# Pooled prevalence of deep vein thrombosis among coronavirus disease 2019 patients

**DOI:** 10.1186/s13054-020-03181-1

**Published:** 2020-07-28

**Authors:** Ying Wang, Li Shi, Haiyan Yang, Guangcai Duan, Yadong Wang

**Affiliations:** 1 Department of Epidemiology, College of Public Health, Zhengzhou University, No. 100 of Science Avenue, Zhengzhou, 450001 China; 2 Department of Toxicology, Henan Center for Disease Control and Prevention, No. 105 of South Nongye Road, Zhengzhou, 450016 China

**Keywords:** Coronavirus disease 2019, Deep vein thrombosis, Prevalence

To the editor,

The article by Ren et al. reported that there was an extremely high incidence (85.4%) of lower extremity deep venous thrombosis (DVT) among 48 patients with severe coronavirus disease 2019 (COVID-19) in Wuhan, China [1]. As the global pandemic of COVID-19, there have been several studies on the incidence, risk factors, and preventive strategies of DVT [1–4]. However, the incidence of DVT has been reported diversely among different clinical centers. Thus, we performed a meta-analysis to estimate the pooled prevalence of DVT in confirmed COVID-19 patients.

We searched PubMed, EMBASE, Web of Science, and medRxiv databases until June 22, 2020, for relevant studies, using the keywords (“coronavirus” or “COVID-19” or “SARS-CoV-2” or “2019-nCoV”) and (“thrombosis” or “thrombi” or “thrombus”). In addition, we screened out the relevant potential articles in the references of selected studies. Articles reporting the prevalence of DVT in confirmed COVID-19 patients were included.

The pooled prevalence and its 95% confidence interval (CI) were used to estimate the combined effects. We calculated the prevalence estimates with the variance stabilizing double arcsine transformation [5, 6]. The heterogeneity among studies was assessed with the *I*^2^ statistic and Cochran’s *Q* test. The meta-regression and subgroup analysis were used to investigate the potential heterogeneity sources (such as sample size, prevalence of prophylaxis in COVID-19 patients, location, design of studies, screening methods of DVT, and COVID-19 patients in intensive care unit (ICU)). We chose Egger’s test and Begg’s test to assess publication bias. All analyses were performed using the Stata 11.2 (StataCorp, College Station, TX), and a two-tailed *P* value < 0.05 was considered to be statistically significant.

A total of 1202 records were initially identified by our searches. We finally included 28 articles in our meta-analysis. The basic characteristics of included studies are shown in Table 1. There were 397 DVT cases in a total of 4138 COVID-19 patients. The pooled estimate of the prevalence for DVT was 16% by using a random-effects model (95% CI 10–23%, *P* < 0.01, *I*^2^ = 96.81, *Q* = 846.41, *P* < 0.01) (Fig. 1a). According to patients’ geographic location, the much higher pooled prevalence of DVT was found in COVID-19 patients from China (30%, 95% CI 2–72%, *P* = 0.02, *I*^2^ = 98.73%, *Q* = 313.90, *P* < 0.01) compared with those from western countries (13%, 95% CI 8–19%, *P* < 0.01, *I*^2^ = 95.62%, *Q* = 502.07, *P* < 0.01) (Fig. 1b). Twenty articles clearly reported the prevalence of DVT in COVID-19 patients treated in ICU or non-ICU. The pooled prevalence of DVT in COVID-19 patients treated in ICU was 23% (95% CI 11–38%, *P* < 0.01, *I*^2^ = 96.44%, *Q* = 421.29, *P* < 0.01), which was significantly higher than in COVID-19 patients treated in non-ICU (5%, 95% CI 1–11%, *P* < 0.01, *I*^2^ = 92.17%, *Q* = 89.42, *P* < 0.01) (Fig. 1c, d). We found significant publication bias by Egger’s test (*P* < 0.001) and Begg’s test (*P* < 0.001). The subgroup analysis showed that none of these factors could explain the significant heterogeneity. However, the meta-regression analysis of multiple covariates indicated that the geographic location of patients could partially explain heterogeneity (*P* = 0.036).

**Table 1:** Characteristics of the included studies

Authors	Sample	Age	Male (%)	Location	Design of studies	Screening of DVT	ICU/non-ICU*	Prophylaxis (%)	DVT (%)
Zhang et al. (PMID: 32421381)	143	63 (mean)	74 (51.7)	China	Cross-sectional study	Ultrasound	N/R	53 (37.1)	66 (46.2)
Ren et al. (PMID: 32412320)	48	70 (median)	26 (54.2)	China	Cross-sectional study	Ultrasound	ICU	47 (97.9)	41 (85.4)
Demelo-Rodríguez et al. (PMID: 32405101)	156	68.1 (mean)	102 (65.4)	Spain	Prospective study	Ultrasound	Non-ICU	153 (98.1)	23 (14.7)
Middeldorp et al. (PMID: 32369666)	198	61 (mean)	130 (65.7)	Netherlands	Retrospective study	Ultrasound	ICU/non-ICU	198 (100)	26 (13.1)
Bi et al.^†^	420	45 (mean)	200 (47.6)	China	Prospective study	N/R	N/R	N/R	6 (1.4)
Klok et al. (PMID: 32291094)	184	64 (mean)	139 (75.5)	Netherlands	Prospective study	Ultrasound	ICU	184 (100)	1 (0.5)
Karmen-Tuohy et al.^‡^	63	61 (mean)	57 (90.5)	USA	Prospective study	N/R	N/R	N/R	2 (3.2)
Llitjos et al. (PMID: 32320517)	26	68 (median)	20 (76.9)	France	Retrospective study	Ultrasound	ICU	8 (30.8)	14 (53.8)
Lodigiani et al. (PMID: 32353746)	388	66 (median)	264 (68.0)	Italy	Retrospective study	Ultrasound	ICU/non-ICU	307 (79.1)	6 (1.7)^§^
Helms et al. (PMID: 32367170)	150	63 (median)	122 (81.3)	France	Prospective study	Imaging	ICU	150 (100)	3 (2.0)
Stoneham et al. (PMID: 32423903)	274	N/R	N/R	UK	Prospective study	Imaging	N/R	N/R	5 (1.8)
Galeano-Valle et al. (PMID: 32425261)	785	N/R	N/R	Spain	Prospective study	Ultrasound	Non-ICU	780 (99.4)	13 (1.7)
Xing et al. (PMID: 32345353)	20	N/R	12 (60.0)	China	Retrospective study	Ultrasound	N/R	N/R	7 (35.0)
Beyls et al. (PMID: 32414510)	12	62 (median)	10 (83.3)	France	Retrospective study	Ultrasound	N/R	N/R	6 (50.0)
Poissy et al. (PMID: 32330083)	107	N/R	N/R	France	Retrospective study	Ultrasound	ICU	107 (100)	5 (4.7)
Beun et al. (PMID: 32311843)	75	N/R	N/R	Netherlands	Retrospective study	N/R	ICU	N/R	3 (4.0)
Cattaneo et al. (PMID: 32349132)	64	70 (median)	35 (54.7)	Italy	Retrospective study	Ultrasound	Non-ICU	64 (100)	0 (0.0)
Tavazzi et al. (PMID: 32322918)	54	N/R	N/R	Italy	Retrospective study	Ultrasound	ICU	54 (100)	8 (14.8)
Voicu et al. (PMID: 32479784)	56	N/R	42 (75.0)	France	Prospective study	Ultrasound	ICU	49 (87.5)	26 (46.4)
Hippensteel et al. (PMID: 32484907)	91	56.5 (mean)	53 (58.2)	USA	Retrospective study	Ultrasound	ICU	N/R	11 (12.1)
Fraissé et al. (PMID: 32487122)	92	61 (median)	73 (79.3)	France	Retrospective study	N/R	ICU	92 (100)	6 (6.5)
Desborough et al. (PMID: 32485437)	66	59 (median)	48 (72.7)	UK	Retrospective study	Imaging	ICU	66 (100)	6 (9.1)
Al-Samkari et al. (PMID: 32492712)	400	61.8 (mean)	228 (57.0)	USA	Retrospective study	Imaging	N/R	400 (100)	10 (2.5)
Edler et al. (PMID: 32500199)	80	79.2 (mean)	46 (57.5)	Germany	Prospective study	N/R	N/R	N/R	32 (40.0)
Grandmaison et al. (PMID: 32529170)	58	N/R	N/R	Switzerland	Cross-sectional study	Ultrasound	ICU/non-ICU	N/R	28 (48.3)
Artifoni et al. (PMID: 32451823)	71	64 (median)	43 (60.6)	France	Retrospective study	Ultrasound	Non-ICU	70 (98.6)	15 (21.1)
Nahum et al. (PMID: 32469410)	34	62.2 (mean)	25 (73.5)	France	Prospective study	Ultrasound	ICU	34 (100)	27 (79.4)
Zhang et al. (PMID: 32553905)	23	44.7 (mean)	15 (65.2)	China	Prospective study	N/R	ICU/non-ICU	N/R	1 (4.3)

*DVT* deep vein thrombosis, *ICU* intensive care unit, *N/R* not (clearly) reported

*Articles clearly reported the prevalence of DVT in COVID-19 patients treated in ICU or non-ICU

^†^doi: 10.1101/2020.04.22.20076190

^‡^doi: 10.1101/2020.05.07.20094797

^§^Data missing for patients

**Fig. 1:**
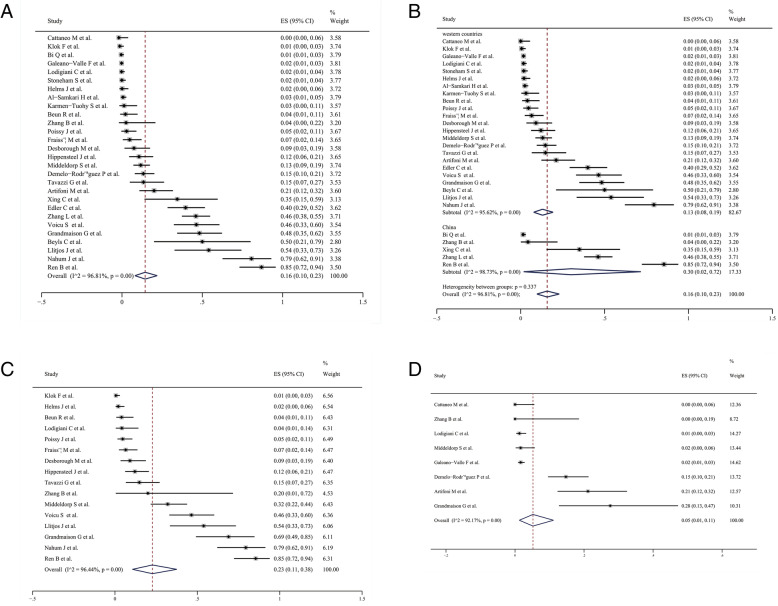
Forest plots of pooled prevalence and its 95% confidence interval (CI) for deep vein thrombosis (DVT) in confirmed coronavirus disease 2019 (COVID-19) patients (**a**) and subgroup analysis by patients’ geographic location (**b**) and the severity of disease (**c**, **d**)

In conclusion, more attention should be paid to the prevention and clinical management of DVT, especially for COVID-19 patients in ICU, and timely assessment of DVT is essential. However, there was considerable heterogeneity in our meta-analysis. In addition, there was significant publication bias in our meta-analysis, although we searched four databases as many and as carefully as possible. Finally, we included non-survival patients who were seriously ill and may exaggerate the prevalence of DVT in COVID-19 patients.

## Data Availability

All data generated or analyzed during this study are included in this article.
